# Energy Status Differentially Modifies Feeding Behavior and POMC^ARC^ Neuron Activity After Acute Treadmill Exercise in Untrained Mice

**DOI:** 10.3389/fendo.2021.705267

**Published:** 2021-06-18

**Authors:** Taylor Landry, Daniel Shookster, Alec Chaves, Katrina Free, Tony Nguyen, Hu Huang

**Affiliations:** ^1^ East Carolina Diabetes and Obesity Institute, East Carolina University, Greenville, NC, United States; ^2^ Department of Kinesiology, East Carolina University, Greenville, NC, United States; ^3^ Human Performance Laboratory, College of Human Performance and Health, East Carolina University, Greenville, NC, United States; ^4^ Department of Physiology, East Carolina University, Greenville, NC, United States

**Keywords:** exercise, food intake, hypothalamus, POMC neuron, NPY/AgRP neuron, tyrosine hydroxylase (th), SIM1 neurons, energy balance

## Abstract

Emerging evidence identifies a potent role for aerobic exercise to modulate activity of neurons involved in regulating appetite; however, these studies produce conflicting results. These discrepancies may be, in part, due to methodological differences, including differences in exercise intensity and pre-exercise energy status. Consequently, the current study utilized a translational, well-controlled, within-subject, treadmill exercise protocol to investigate the differential effects of energy status and exercise intensity on post-exercise feeding behavior and appetite-controlling neurons in the hypothalamus. Mature, untrained male mice were exposed to acute sedentary, low (10m/min), moderate (14m/min), and high (18m/min) intensity treadmill exercise in a randomized crossover design. Fed and 10-hour-fasted mice were used, and food intake was monitored 48h. post-exercise. Immunohistochemical detection of cFOS was performed 1-hour post-exercise to determine changes in hypothalamic NPY/AgRP, POMC, tyrosine hydroxylase, and SIM1-expressing neuron activity concurrent with changes in food intake. Additionally, stains for pSTAT3^tyr705^ and pERK^thr202/tyr204^ were performed to detect exercise-mediated changes in intracellular signaling. Results demonstrated that fasted high intensity exercise suppressed food intake compared to sedentary trials, which was concurrent with increased anorexigenic POMC neuron activity. Conversely, fed mice experienced augmented post-exercise food intake, with no effects on POMC neuron activity. Regardless of pre-exercise energy status, tyrosine hydroxylase and SIM1 neuron activity in the paraventricular nucleus was elevated, as well as NPY/AgRP neuron activity in the arcuate nucleus. Notably, these neuronal changes were independent from changes in pSTAT3^tyr705^ and pERK^thr202/tyr204^ signaling. Overall, these results suggest fasted high intensity exercise may be beneficial for suppressing food intake, possibly due to hypothalamic POMC neuron excitation. Furthermore, this study identifies a novel role for pre-exercise energy status to differentially modify post-exercise feeding behavior and hypothalamic neuron activity, which may explain the inconsistent results from studies investigating exercise as a weight loss intervention.

## Introduction

The hypothalamus is a critical nexus of neuron populations that interpret peripheral signals of energy status and deliver diverse efferent outputs to metabolically active tissues (1, 2). These neurons are critical to maintaining metabolic homeostasis, and disruption of their complex neurocircuitry is associated with disordered substrate metabolism and feeding behavior (1, 2). Additionally, emerging evidence identifies a potent role for aerobic exercise to modulate activity and synaptic organization of hypothalamic neurons, especially in the arcuate nucleus (ARC) (3–5). This hypothalamic region contains diverse neuron populations involved in regulating appetite; thus, the ARC presents an attractive target to investigate the regulation of post-exercise feeding behavior.

Identified ARC neurons modulated by acute treadmill exercise include the neuropeptide Y/agouti-related peptide (NPY/AgRP^ARC^) and pro-opiomelanocortin (POMC^ARC^) -expressing neuron populations (3–7). POMC^ARC^ neurons suppress food intake by releasing α-melanocyte-stimulating hormone (αMSH), which binds to melanocortin 4 receptors (MC4R’s) to excite satiety-inducing neurons in the paraventricular nucleus of the hypothalamus (PVN) (1, 8). NPY/AgRP^ARC^ neurons have opposite effects on MC4R-expressing neuron activity and feeding *via* co-release of gamma aminobutyric acid (GABA), NPY, and AgRP (1, 2, 9). NPY/AgRP neurons may also directly antagonize POMC neurons *via* GABAergic connections, but the relevance of this phenomenon in physiological conditions is unclear (10).

Both NPY/AGRP^ARC^ and POMC^ARC^ neurons are subject to intricate regulation by appetite-stimulating tyrosine hydroxylase neurons in the ARC (TH^ARC^) (11). TH is the rate-limiting enzyme in dopamine synthesis (12), and the effects of exercise on TH^ARC^ neurons have yet to be investigated. TH^ARC^ neurons exhibit direct excitatory dopaminergic innervation on NPY/AgRP^ARC^ neurons, as well as direct inhibitory dopaminergic and GABAergic innervation on POMC^ARC^ neurons (11). Moreover, another subpopulation of TH-expressing neurons resides in the PVN (TH^PVN^) and receives presynaptic inputs from NPY/AgRP^ARC^ neurons to stimulate thermogenesis in brown adipose tissue (13). To date, the potential role of TH^PVN^ neurons in appetite regulation is unknown.

ARC neurons are located adjacent to the third ventricle and the median eminence, which provides convenient access to the cerebrospinal fluid (CSF) and a less selective blood-brain barrier, respectively, and allows for fine responsiveness to changes in energy status. For example, to promote feeding in response to fasting, ghrelin concentrations increase (14), while leptin and insulin levels decrease (15, 16), directly resulting in receptor-mediated increases in NPY/AgRP neuron activity and decreases in POMC neuron activity (17–20). Reduced glucose levels have similar effects on NPY/AgRP and POMC neurons; during which, glucose uptake by glucose transporters is reduced and activity of ATP-sensitive potassium channels is altered (21, 22). Glucose, insulin, leptin, and ghrelin concentrations also differentially fluctuate in response to exercise, depending on intensity and duration (4, 23–25); thus, insight into exercise-mediated changes in hypothalamic neuron activity could be beneficial to understanding of the complex physiological mechanisms regulating post-exercising feeding behavior.

Studies investigating exercise-mediated remodeling of hypothalamic neurocircuits produce conflicting results. For example, Bunner et al. (4) observed acute moderate intensity treadmill exercise (MIE) to increase NPY/AgRP^ARC^ neuron activity and subsequent food intake in fed mice, while He et al. (5) demonstrated opposite effects after fed high intensity interval training (HIIT). Results in POMC^ARC^ neurons are equally equivocal, with one report observing no changes in response to fasted MIE (4) and others observing increased activity after fasted high intensity exercise (HIE) (3) and fed HIIT (5). Notably, all these studies analyzed neuronal activity at a single timepoint immediately after exercise, and exercise-mediated changes in neuronal activity in the hypothalamus can be rapid and transient (5–7). The reported effects may reflect neuroendocrine responses during exercise, rather than changes after exercise, and may also miss critical changes in neuronal activity in the hours after exercise. Moreover, the studies observing appetite-suppressing, NPY/AgRP neuron inhibiting, and/or POMC activating effects after treadmill exercise used electric shock to motivate mice to run, which was not controlled for in sedentary trials (3, 5). This may be a confounding factor, since studies have demonstrated electric shock activates satiety-inducing neurons in the PVN and acute stress stimulates POMC^ARC^-mediated hypophagia (26–28). Alternatively, He et al. (5) may have identified a unique appetite-suppressing effect specific to HIIT. Supporting this hypothesis, other studies have also observed decreased food intake in response to voluntary wheel running in rodents (29, 30), which typically is a more intermittent exercise model (31).

Another potential explanation for variability in investigations into exercise-mediated changes in hypothalamic neuron activity is that these studies contain methodological differences in exercise intensity and pre-exercise feeding (3–5). Considering glucose and metabolic hormone concentrations fluctuate with energy status and exercise intensity (19, 20, 22, 32), it is plausible that post-exercise changes in hypothalamic neuron activity vary depending on energy status and exercise intensity as well. For example, a recent report demonstrates that NPY/AgRP neurons are activated during fed HIIT, but exhibit opposite effects during fasted HIIT (6). Furthermore, the magnitude of these changes in neuronal activity is directly correlated with exercise intensity (6). Notably, these observations were made during exercise and it remains to be determined if post-exercise changes in hypothalamic neuron activity are similarly dynamic depending on energy status or exercise intensity. This potential phenomenon may explain why the effects of exercise on feeding behavior are inconsistent in the literature and why the success rates of exercise as a weight loss intervention are equally unpredictable (33–44). Overall, the potential confounding factors and methodological differences in studies investigating exercise-mediated modulation of hypothalamic neurons make drawing conclusions challenging; thus, the current study aimed to utilize a translational, well-controlled, within-subject, treadmill exercise protocol to determine the differential effects of fed *vs.* fasted exercise and exercise intensity on subsequent feeding behavior and hypothalamic neuron activity.

## Materials and Methods

### Animals

Male B6.Tg(NPY-hrGFP)1Lowl/J (NPY-GFP reporter) mice were cared for in accordance with the National Institutes of Health *Guide for the Care and Use of Laboratory Animals*, and experimental protocols were approved by Institutional Animal Care and Use Committee of East Carolina University. Mice were fed standard chow ad libitum (3.2kcal/g) and housed at 20–22°C with a 12-h light-dark cycle.

### High-Fat Diet-Induced Obesity

5-6-week-old NPY-GFP reporter mice were given ad libitum access to a high-fat diet (5.56kcal/g) with a kilocalorie composition of 58%, 25%, and 17% of fat, carbohydrate, and protein, respectively, for 10 weeks (D12331; Research Diets, New Brunswick, NJ) before undergoing exercise trials.

### Acute Treadmill Exercise

Exercise protocols were adapted from a previous study in our lab (4) and all mice were able to successfully complete all exercise trials. Briefly, using a randomized crossover design and commonly used treadmill speeds, untrained mice performed 1-hour sedentary, low (10m/min) (45), moderate (14m/min) (46), and high intensity (18m/min) (3) exercise with one week between bouts. On the day before experiments, mice were familiarized by running for 5 min at 5m/min followed by 5 min at 10m/min. On the day of exercise trials, mice ran at 5m/min for two minutes, 10m/min for two minutes, and then, when applicable, sped up 4m/min every 2 min until the target speed was reached. To minimize stress, a soft bristle brush or gentle puff of air was used to motivate mice, when needed. During sedentary trials, mice were placed in empty cages on top of the running treadmill for 1-hour to simulate stress during exercise bouts. For fasting exercise experiments, food was removed from cages 10-hours before exercise. All sedentary and exercise bouts were performed between 6:30 and 7:30pm., immediately before the dark-phase.

### Food Intake and Body Weight Measurements

Food was weighed and added to individually-housed cages immediately after sedentary and exercise bouts (14-15g). Measurements were made 1, 2, 3, 6, 12, 24, and 48-hours post-exercise by subtracting from the total food. Bedding was inspected thoroughly for residual bits of food, which were included in measurements. All food intakes were normalized to body weight, which was measured immediately before sedentary and exercise bouts.

### Immunohistochemistry

1-hour post-exercise, when differences in food intake were first observed, mice were intracardially perfused with PBS followed by 10% formalin before immunohistochemistry was performed as described previously (47). Briefly, brains were sliced into 20-μm coronal sections using a freezing microtome (VT1000 S; Leica, Wetzlar, Germany) and incubated overnight in antibody to cFOS (1:250; Santa Cruz Biotechnology, Santa Cruz, CA), POMC (1:6000; Phoenix Pharmaceuticals), tyrosine hydroxylase (TH) (1:100000; Millipore), and SIM1 (1:250; Millipore) followed by incubation with Alexa-fluorophore secondary antibody for 1 h (Abcam). To probe for two proteins with same-source antibodies DAB (3,3′-Diaminobenzidine) staining was performed prior to the immunohistochemistry protocols described above (4). Briefly, brain sections were incubated overnight in antibody to phosphorylated STAT3^tyr705^ (1:500; Cell Signaling Technology) or phosphorylated ERK^thr202/tyr204^ (1:500; Cell Signaling Technology) followed by biotinylated donkey anti-rabbit IgG (Vector; 1:1000) for 2 hours. Sections were then incubated in the avidin–biotin complex (ABC; Vector Elite Kit; 1:500) and incubated in 0.04% DAB, 0.02% cobalt chloride (Fisher Scientific), and 0.01% hydrogen peroxide. Note, pSTAT3^tyr705^ images were inverted (to white) for colocalization. All stains were photographed using an optical microscope (DM6000; Leica), followed by blind analysis using ImageJ. At least three anatomically matched images per mouse were quantified.

### Statistical Analysis

For time course food intake experiments, differences among sedentary or exercise bouts were determined using 2-Way-ANOVA with Repeated Measures for time and treatment (n=11/group). Bonferroni corrections were used for multiple comparisons. For comparisons in neuronal activity between sedentary and high intensity exercise, Student’s T-tests were used (n=5-7/group for POMC^ARC^ neurons, 4-6/group for SIM1^PVN^ neurons, 5-6/group for TH^ARC^ neurons, 3-5/group for TH^PVN^ neurons, and 4-6/group for NPY/AgRP^ARC^ neurons). All analyses were performed using GraphPad Prism statistics software, and *p*<0.05 was considered statistically significant.

## Data Availability Statement

The data sets generated during the current study are available from the corresponding author on reasonable request.

## Results

### Acute High Intensity Treadmill Exercise Suppresses Food Intake in Fasted Mice

**Figure 1:**
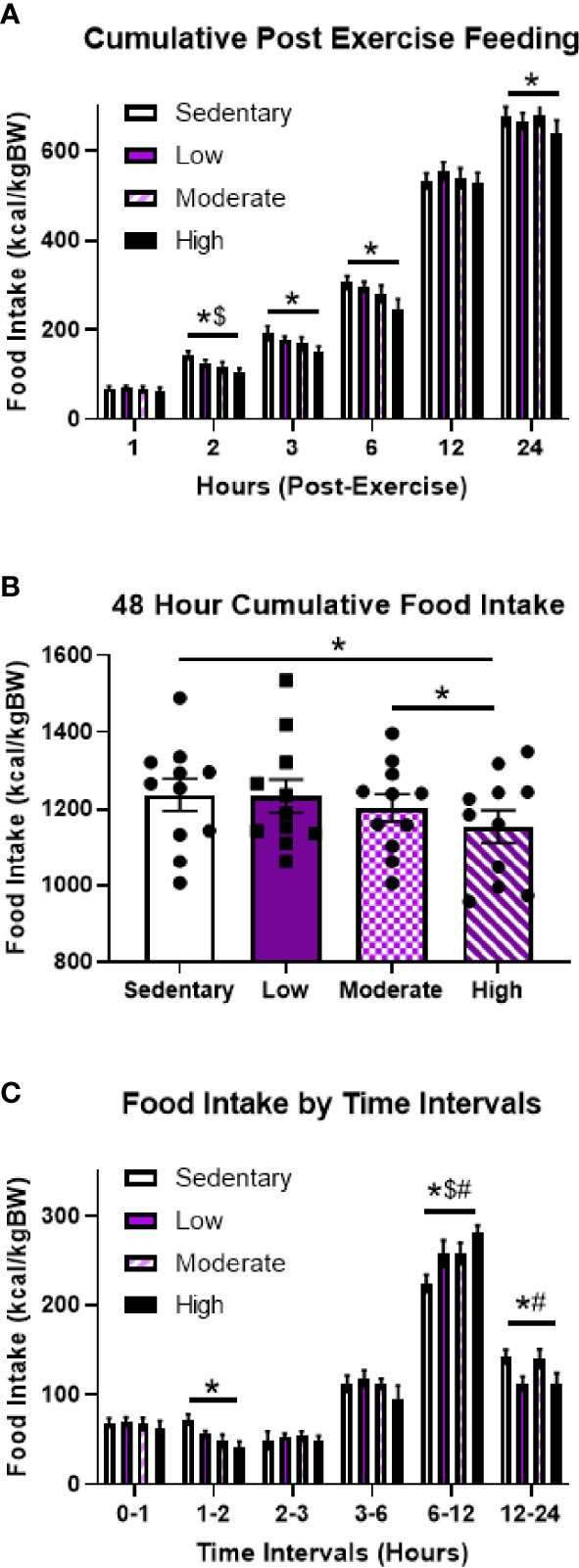
Acute high intensity treadmill exercise suppresses food intake in fasted male mice. **(A)** Timeline of cumulative food intake, **(B)** 48-hour cumulative food intake, and **(C)** food intake by time intervals in fasted male mice in response to different acute treadmill exercise intensities (n = 11). Data represented as mean ± SEM. *indicates p < 0.05 high intensity *vs.* sedentary; ^$^indicates p < 0.05 moderate intensity *vs.* sedentary; ^#^indicates p < 0.05 low intensity *vs.* sedentary.

To investigate the effects of varying aerobic exercise intensities on post-exercise food intake, 11 fasted male mice underwent acute sedentary, low intensity (LIE), MIE, and HIE bouts for 1-hour on the treadmill. Cumulative 24-hour food intake was unchanged after LIE and MIE; however, mice ate significantly less after HIE compared to sedentary bouts (5.3%), which persisted for at least 48 hours (6.7%) (**Figures 1A, B**). Further examination of food intake at specific time intervals revealed HIE-mediated appetite suppression occurred 1-2 hours post-exercise, followed by a compensatory increase in food intake at 6-12 hours, and another period of significantly reduced food intake at 12-24 hours (**Figure 1C**). MIE also elicited trends toward reduced food intake 1-2 hours post-exercise, but these effects were not statistically significant (p=0.16), and all exercise intensities elicited increases in food intake at 6-12 hours. Taken together, these data suggest HIE uniquely results in a suppression in appetite 1-2 and 12-24-hours post-exercise, resulting in reduced cumulative food intake for at least 48 hours.

Surprisingly LIE and HIE had no effects on food intake in DIO mice, but MIE significantly reduced 24-hour food intake 8.5% (**Supplemental Figure 1A**) and trended to decrease cumulative food intake at 48 hours (p=0.10; **Supplemental Figure 1B**). Specifically, the reductions in food intake in response to MIE were predominantly observed 2-3 hours post-exercise (**Supplemental Figure 1C**). In summary, these findings suggest, even in overweight mice, fasted aerobic exercise can have an appetite-suppressing effect.

### High Intensity Exercise Increases Activity of POMC^ARC^ and SIM1^PVN^ Neurons in Fasted Mice

**Figure 2:**
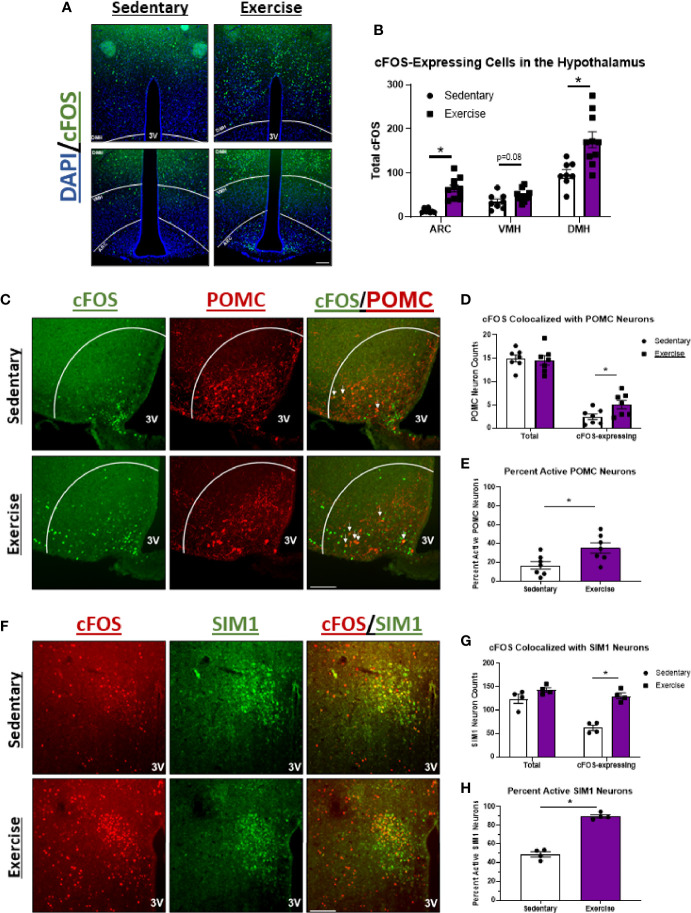
POMC^ARC^ and SIM1^PVN^ neuron activity is elevated 1 hour after acute high intensity treadmill exercise in fasted male mice. **(A)** Representative images of cFOS (green) in the hypothalamus of fasted male mice 1 hour after sedentary trials or high intensity treadmill exercise (DAPI in blue). **(B)** Total cFOS-expressing cells in each region of the hypothalamus (n=10/group) DMH=dorsomedial hypothalamus; VMH=ventromedial hypothalamus; ARC=arcuate nucleus. **(C)** Representative images of cFOS (green) colocalized with POMC^ARC^ neurons (red) in fasted male mice 1 hour after sedentary trials or high intensity treadmill exercise. **(D)** Total and cFOS-expressing POMC^ARC^ neurons and **(E)** Percent active POMC^ARC^ neurons (n = 7/group). **(F)** Representative images of cFOS (red) colocalized with SIM1^PVN^ neurons (green) in fasted male mice 1 hour after sedentary trials or high intensity treadmill exercise. **(G)** Total and cFOS-expressing SIM1^PVN^ neurons and **(H)** Percent active SIM1^PVN^ neurons (n = 4/group). 3V = Third ventricle; Scale bar = 50um. Data represented as mean ± SEM. *indicates p < 0.05 *vs.* sedentary trials.

Since HIE was the only bout that elicited effects on food intake, brains were removed 1-hour after HIE and immunohistochemical detection for cFOS (a marker of neuronal activity) was performed in the hypothalamus to investigate the associated changes in neuronal activity. Compared to sedentary controls, HIE significantly increased cFOS expression in the dorsomedial hypothalamus (1.8-fold) and ARC (4.4-fold), while trending to increase cFOS in the ventromedial hypothalamus (1.4-fold; p=0.08) (**Figures 2A, B**). Double-staining for cFOS and POMC-expressing neurons revealed that the robust increase in ARC cFOS post-exercise is, in part, due to POMC^ARC^ neuron excitation (2-fold) (**Figures 2C–E**), which are well-documented appetite-suppressing neurons (8). Additionally, HIE increased cFOS colocalization with SIM1^PVN^-expressing neurons 2.1-fold, which are also satiety-inducing neurons with presynaptic input from POMC neurons (**Figures 2F–H**) (2, 48). These data suggest that the POMC^ARC^→ SIM1^PVN^ neurocircuit may be activated by HIE.

### High Intensity Exercise Also Increases Activity of NPY/AgRP^ARC^ and TH^PVN^ Neurons in Fasted Mice

**Figure 3:**
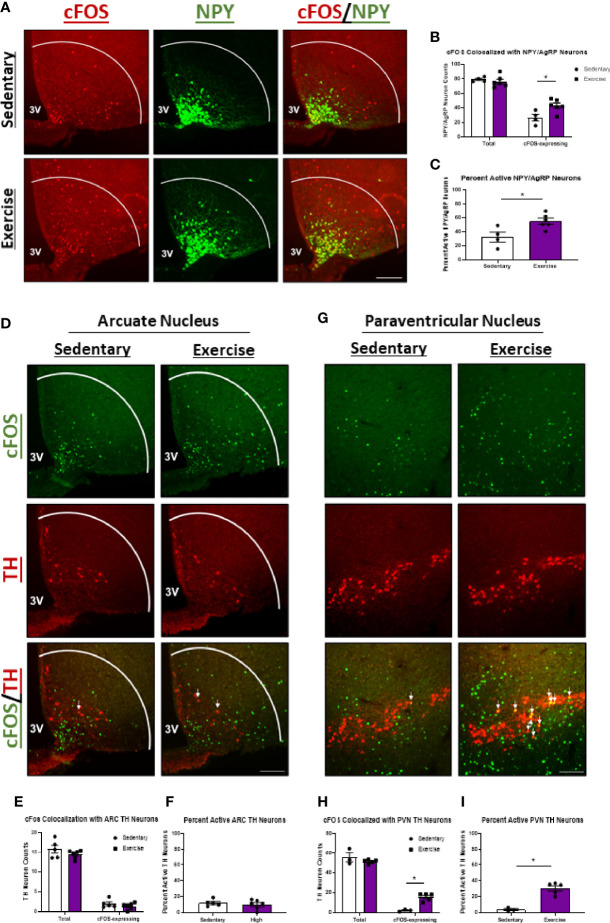
NPY/AgRP^ARC^ and TH^PVN^ neuron activity is elevated 1 hour after acute high intensity treadmill exercise in fasted male mice. **(A)** Representative images of cFOS (red) colocalized with NPY/AgRP^ARC^ neurons (green) in fasted male mice 1 hour after sedentary trials or high intensity treadmill exercise. **(B)** Total and cFOS-expressing NPY/AgRP^ARC^ neurons and **(C)** Percent active NPY/AgRP^ARC^ neurons (n = 4-6/group). **(D)** Representative images of cFOS (green) colocalized with TH^ARC^ neurons (red) in fasted male mice 1 hour after sedentary trials or high intensity treadmill exercise. **(E)** Total and cFOS-expressing TH^ARC^ neurons and **(F)** Percent active TH^ARC^ neurons (n = 5-6/group). **(G)** Representative images of cFOS (green) colocalized with TH^PVN^ neurons (red) in fasted male mice 1 hour after sedentary trials or high intensity treadmill exercise. **(H)** Total and cFOS-expressing TH^PVN^ neurons and **(I)** Percent active TH^PVN^ neurons (n = 3-5/group). 3V = Third ventricle; Scale bar = 50um. Data represented as mean ± SEM. *indicates p < 0.05 *vs.* sedentary trials.

We also investigated the effects of HIE on activity of appetite-inducing NPY/AgRP^ARC^ and TH^ARC^ neurons. Surprisingly, cFOS colocalization with NPY/AgRP^ARC^ neurons was elevated 1.7-fold 1-hour post-HIE (**Figures 3A–C**). While these data are conflicting with the appetite-suppressing effects of HIE, previous reports suggest exercise-mediated increases in NPY/AgRP neuron activity may be critical to ensuring adequate post-exercise refueling and recovery (4). Moreover, HIE had no effects on cFOS colocalization with TH^ARC^ neurons (**Figures 3D–F**); however, cFOS expression in TH^PVN^ neurons was increased 8.6-fold (**Figures 3G–I**).

The diverse changes in neuronal activity in response to HIE occurred independent from changes in ARC pSTAT3^tyr705^ and pERK^thr202/tyr204^ intracellular signaling 1-hour post-exercise (**Supplemental Figure 2**). pSTAT3^tyr705^ and pERK^thr202/tyr204^ are downstream mediators of the anorexigenic hormone leptin to activate POMC neurons and inhibit NPY/AgRP neurons (17, 49); therefore, the appetite suppressing effects of HIE may be independent from leptin activity.

### Aerobic Exercise in the Fed State Increases Post-Exercise Food Intake

While our data in fasted mice suggests HIE would be beneficial to creating a caloric deficit by increasing energy expenditure and decreasing food intake, recent studies investigating the effects of aerobic exercise on feeding behavior and appetite-controlling neurons produce conflicting results (3–5). Since the hypothalamic neurons affected by aerobic exercise are sensitive to changes in glucose and metabolic hormones (19, 20, 22, 32), we hypothesized that changes in pre-exercise energy status may differentially modulate the post-exercise feeding response. Compared to 10-hour-fasted sedentary mice, a separate cohort of ad libitum fed sedentary mice exhibited significantly elevated cFOS colocalization with POMC^ARC^ neurons (3.3-fold) (**Supplemental Figure 3A**) and trends toward decreased cFOS colocalization with NPY/AgRP^ARC^ neurons (p=0.08; 0.6-fold) (**Supplemental Figure 3B**). Moreover, both 1-hour and 24-hour cumulative food intake was decreased in fed mice, overall validating significant differences in pre-exercise energy status between fasted and fed mice (**Supplemental Figure 3C**).

**Figure 4:**
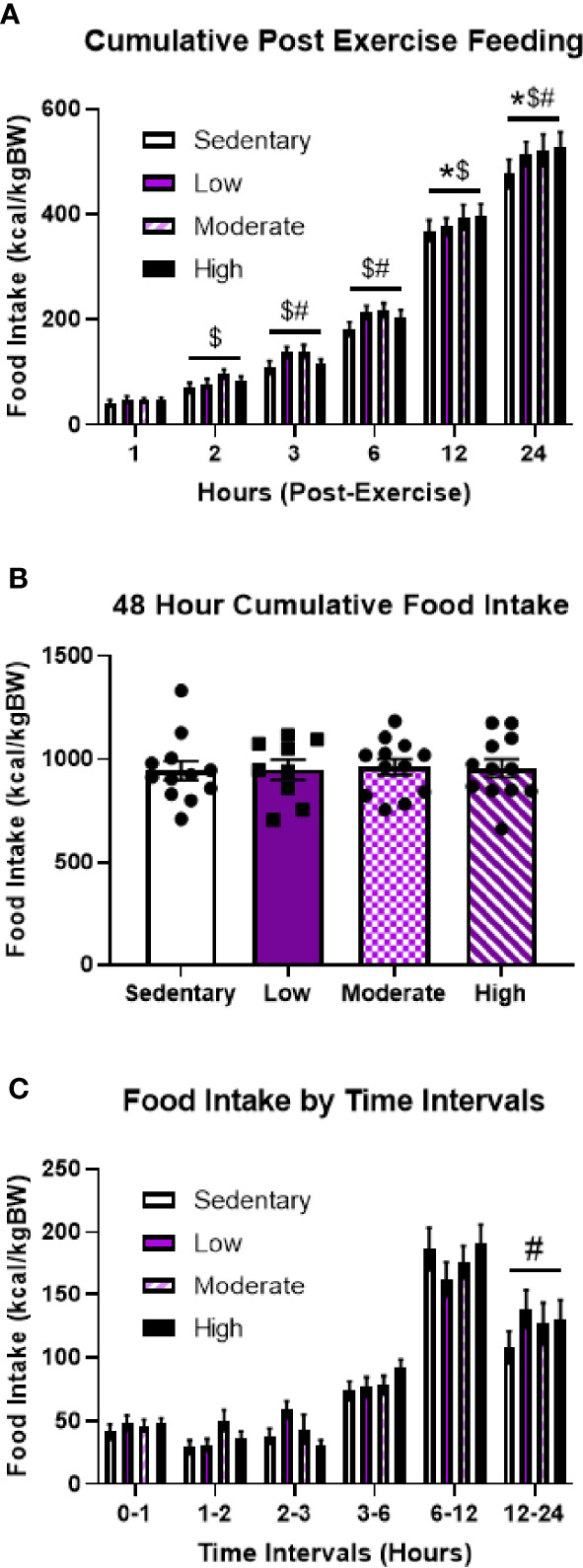
Regardless of intensity, acute treadmill exercise increases food intake in fed male mice. **(A)** Timeline of cumulative food intake, **(B)** 48-hour cumulative food intake, and **(C)** food intake by time intervals in fed male mice in response to different acute treadmill exercise intensities (n = 11). Data represented as mean ± SEM. *indicates p < 0.05 high intensity *vs.* sedentary; ^$^indicates p < 0.05 moderate intensity *vs.* sedentary; ^#^indicates p < 0.05 low intensity *vs.* sedentary.

Supporting the hypothesis that pre-exercise energy status dictates post-exercise feeding behavior, all exercise intensities resulted in 8-10% increases in 24-hour food intake compared to the sedentary bout (**Figure 4A**). Notably, these differences were no longer evident after 48 hours (**Figure 4B**). Analysis of specific time intervals revealed that LIE specifically elicited increased food intake between 12 and 24 hours post-exercise; however, there were no significant differences at any specific time intervals in response to MIE and HIE (**Figure 4C**). Overall, while time interval data suggests heterogeneity in the specific post-exercise feeding timelines among fed mice, these findings highlight drastic differences in post-exercise feeding behavior depending on energy status.

### POMC^ARC^ Neuron Excitation Is Specific to High Intensity Exercise in the Fasted Status

**Figure 5:**
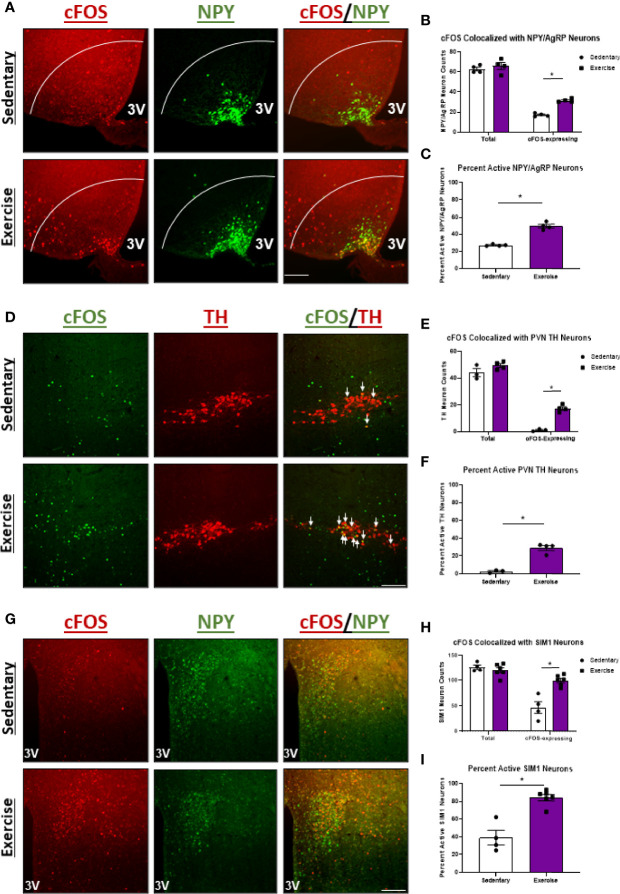
Regardless of energy status, high intensity treadmill exercise increases NPY/AgRP^ARC^, TH^PVN^, and SIM1^PVN^ neuron activity. **(A)** Representative images of cFOS (red) colocalized with NPY/AgRP^ARC^ neurons (green) in male mice 1 hour after sedentary trials or high intensity treadmill exercise. **(B)** Total and cFOS-expressing NPY/AgRP^ARC^ neurons and **(C)** Percent active NPY/AgRP^ARC^ neurons (n=4/group). **(D)** Representative images of cFOS (green) colocalized with TH^PVN^ neurons (red) in fed male mice 1 hour after sedentary trials or high intensity treadmill exercise. **(E)** Total and cFOS-expressing TH^PVN^ neurons and **(F)** Percent active TH^PVN^ neurons (n = 3-4/group). **(G)** Representative images of cFOS (red) colocalized with SIM1^PVN^ neurons (green) in fed male mice 1 hour after sedentary trials or high intensity treadmill exercise. **(H)** Total and cFOS-expressing SIM1^PVN^ neurons and **(I)** Percent active SIM1^PVN^ neurons (n = 4-6/group). 3V, Third ventricle; Scale bar = 50um. Data represented as mean ± SEM. *indicates p < 0.05 *vs.* sedentary trials.

**Figure 6:**
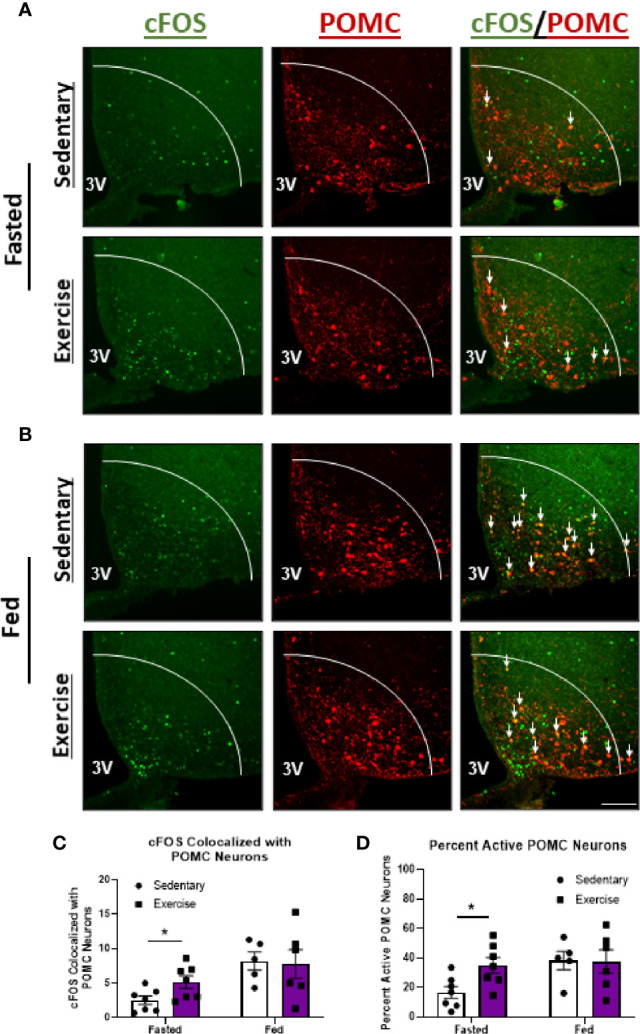
Exercise-mediated POMC^ARC^ activation is specific to the fasted status. Representative images of cFOS (green) colocalized with POMC^ARC^ neurons (red) in **(A)** fasted or **(B)** fed male mice 1 hour after sedentary trials or high intensity treadmill exercise. **(C)** cFOS-expressing POMC^ARC^ neurons and **(D)** Percent active POMC^ARC^ neurons (n = 5-7/group). 3V = Third ventricle; Scale bar = 50um. Data represented as mean ± SEM. *indicates p < 0.05 *vs.* sedentary trials.

We next aimed to compare changes in hypothalamic neuron activity between exercise in the fasted and fed statuses to better understand the neuron populations responsible for energy status-dependent feeding behavior. Similar to HIE in the fasted status, HIE in fed mice resulted in increased cFOS expression in NPY/AgRP^ARC^ (1.8-fold; **Figures 5A–C**), TH^PVN^ (11.4-fold; **Figures 5D–F**), and SIM1^PVN^ (2.2-fold; **Figures 5G–I**) neurons, with no effects in TH^ARC^ neurons (**Supplemental Figure 4**). The similarities in hypothalamic NPY/AgRP, TH, and SIM1 neuron responses between fed and fasted exercise indicate these neuron populations may not be responsible for energy status-dependent effects of exercise on feeding behavior. Interestingly, unlike in fasted mice, fed HIE had no effects on cFOS colocalization with POMC^ARC^ neurons (**Figure 6**), suggesting POMC excitation may be, in part, responsible for energy status-dependent variations in post-exercise feeding.

## Discussion

Recent studies identifying a potent ability for aerobic exercise to modulate hypothalamic neurons involved in appetite regulation produce conflicting results (3–5). These discrepancies may be explained by differences in methods utilized, including exercise intensity, pre-exercise energy status, and stimulus to promote the exercise. Considering this, the current study used a translational, within-subject, acute treadmill exercise protocol to determine that pre-exercise energy status and exercise intensity drastically modify post-exercise feeding behavior and concurrent hypothalamic neuron activity.

The current study identified a beneficial role of fasted aerobic exercise to suppress food intake in mice, which is consistent with some previous reports in rodents and humans (3, 35, 40, 50, 51). Interestingly, reduced food intake occurred only after HIE in lean mice, whereas in DIO mice, this effect was limited to the MIE bout. At first glance, this suggests DIO mice have a lower intensity threshold to elicit exercise-mediated reductions in food intake; however, intensities used in the current study are based on absolute treadmill speeds and do not account for intrinsic aerobic capacities or differences in body weight. Considering DIO mice have lower aerobic capacities compared to lean mice (52), and greater body mass requires more work to perform the same treadmill exercise, the relative intensity at which DIO mice are exercising is higher at any given absolute treadmill speed. Therefore, it is plausible that the HIE and MIE are eliciting similar relative intensities for the lean and DIO mice, respectively. Consequently, these findings suggest fasted aerobic exercise at higher intensities can reduce food intake regardless of obesity, and, coupled with the associated energy expenditure, may be optimal for creating a caloric deficit and inducing weight loss.

Despite our encouraging evidence suggesting higher intensity exercise can suppress appetite, the literature regarding post-exercise feeding produces inconsistent results (35, 36, 39–44, 50, 51, 53–55). The current study demonstrated a novel phenomenon in which post-exercise feeding behavior is dependent on pre-exercise energy status, which may explain these discrepancies. For example, fasted HIE suppressed food intake in lean mice, but fed exercise increased food intake regardless of intensity. While the reasons for these energy status-dependent differences in feeding behavior are unclear, glucose and metabolic hormone concentrations also fluctuate with energy status, modulating activity of neuron populations involved in regulating feeding behavior (19, 20, 22, 32). Consequently, recent studies have investigated the potential role for exercise to modulate activity of hypothalamic neurons involved in regulating appetite (3–7).

To elucidate the neuronal mechanisms underlying energy status-dependent post-exercise feeding, we compared the changes in hypothalamic neuron activity after fasted *vs.* fed HIE. The primary difference observed was an increase in anorexigenic POMC^ARC^ neuron activity 1-hour after fasted, but not fed, HIE. Since POMC^ARC^ neurons suppress food intake through excitatory synaptic connections with MC4R^PVN^-expressing neurons (1, 8), these results may suggest POMC^ARC^ neurons are a mediating factor in fasted HIE-mediated appetite suppression; although at this time it is unclear what is driving these energy status dependent changes. Feeding increases POMC^ARC^ neuron activity (1, 56); therefore it’s possible POMC^ARC^ neuron activity has plateaued before fed exercise, resulting in no additive effects of exercise on these neurons. Mechanistically, exercise has been shown to increase sensitivity to the POMC^ARC^-stimulating hormone leptin (24, 57); however our data revealed no changes in the ARC in pSTAT3^tyr705^ or pERK^thr202/tyr204^, which are key mediators of leptin activity. Ropelle et al. (57) also observed no effects of exercise on hypothalamic pSTAT3^tyr705^ in lean mice, despite observing augmented appetite suppression in response to intracerebroventricular (ICV) leptin infusion. Since higher intensity exercise can decrease leptin levels (24), the absence of changes in downstream leptin signaling in the hypothalamus post-exercise may suggest compensatory increases in leptin sensitivity. Alternatively, exercise elevates IL-6 concentrations (58), improves insulin sensitivity (57), increases core temperature (3), and decreases ghrelin levels (59), which all can increase POMC^ARC^ neuron activity. Future studies investigating differential modulation of hormones and core temperature by fasted *vs.* fed exercise may provide further explanation into energy-status dependent POMC^ARC^ activation.

While our POMC^ARC^ results are consistent with past reports observing fasting HIE increases POMC^ARC^ neuron activity (3) and no effects of MIE in fed mice (4), one study discovered HIIT has excitatory effects on POMC^ARC^ neurons, even in the fed state (5). It is possible HIIT elicits additional appetite-suppressing benefits compared to steady-state HIE, independent from energy status. This hypothesis is supported by studies observing voluntary wheel running, which is a more intermittent model of exercise (31), to also suppress food intake (29, 30). Alternatively, He et al. (5) utilized electric shock as a stimulus to motivate mice during exercise, which may have been a confounding factor. Electric shock has been shown to activate satiety-inducing neurons in the hypothalamus (28), and acute stress in mice promotes POMC^ARC^-mediated hypophagia (26, 27). These confounding factors highlight the importance of minimizing and controlling for stress in these studies.

Increased activity of many other neuron populations in the hypothalamus was observed in the present study in response to HIE. For example, NPY/AgRP^ARC^ and SIM1^PVN^ neuron activity was elevated regardless of pre-exercise energy status, suggesting these neuron populations are not driving energy status-dependent feeding behavior after exercise. Activation of the orexigenic NPY/AgRP^ARC^ neuron population was surprising, although a previous study demonstrated NPY/AgRP^ARC^ activation post-exercise was essential to refeeding (4). Therefore, exercise-mediated NPY/AgRP^ARC^ neuron activation may be an evolutionarily preserved mechanism to ensure adequate refueling and recovery. Conversely, He et al., 2018 (5) observed inhibitory effects of HIIT on NPY/AgRP^ARC^ neurons, however, as previously mentioned, these effects may be unique to HIIT or a biproduct of electric shock-induced stress. While the importance of exercise-mediated increases in SIM1^PVN^ neuron activity to feeding behavior is unclear, these findings were consistent with a previous study (4). Past reports using chemogenetic approaches determined exercise-induced SIM1^PVN^ activity is independent from presynaptic NPY/AgRP^ARC^ neurons (4); however, recently a subpopulation of SIM1^PVN^ neurons that exhibit excitatory synapses on NPY/AgRP^ARC^ neurons was identified (60). It is possible that aerobic exercise stimulates a SIM1^PVN^→NPY/AgRP^ARC^ neurocircuit to promote adequate refeeding, and future studies using chemogenetic inhibition of SIM1^PVN^ neurons before aerobic exercise could help verify this hypothesis.

To our knowledge, the present study was the first to investigate the effects of HIE on hypothalamic TH-expressing neurons. TH^ARC^ neurons stimulate food intake by releasing GABA and dopamine (11); however, no effects of exercise on TH^ARC^ neuron activity were observed. Interestingly, a striking increase in TH^PVN^ neuron activity was observed in response to both fed and fasted exercise. TH^PVN^ neurons have been implicated in the regulation of brown adipose tissue thermogenesis (13), and they exhibit downstream synaptic connections in various areas of the hypothalamus (61), but their role in appetite regulation is unknown. Regardless, elevated TH^PVN^ neuron activity in response to both fed and fasted exercise suggests these neurons are also not driving factors in energy status-dependent feeding behavior post-exercise.

**Figure 7:**
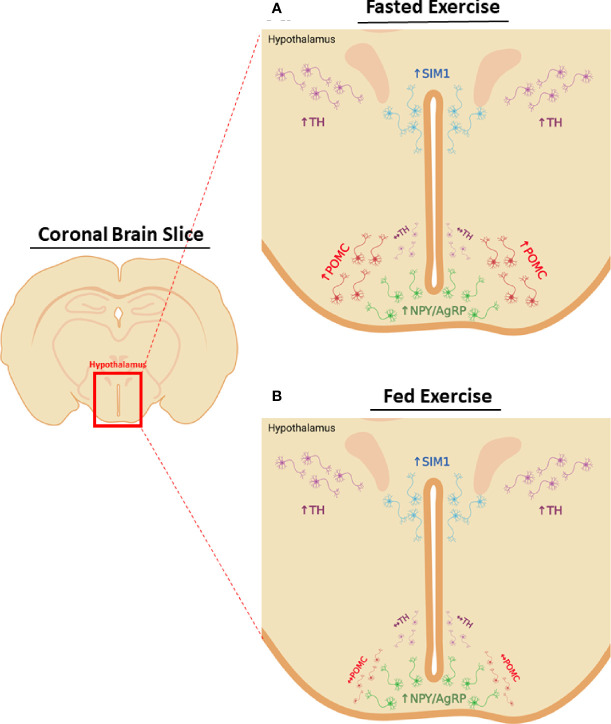
Energy status differentially modifies post-exercise neuronal activity in the hypothalamus. Changes in hypothalamic neuron activity 1-hour after HIE in the **(A)** fasted or **(B)** fed status. Regardless of energy status HIE increases NPY/AgRP^ARC^, SIM1^PVN^, and TH ^PVN^ neuron activity, with no effects on TH^ARC^ neurons. POMC^ARC^ neuron activity is only elevated 1-hour after fasted HIE.

To summarize the neuronal phenotype in response to acute HIE, NPY/AgRP^ARC^, TH^PVN^, and SIM1^PVN^ neuron activity are elevated 1-hour post-exercise, regardless of energy status, with no effects on TH^ARC^ neurons. Surprisingly, POMC^ARC^ activity is only increased after fasted exercise, suggesting these neurons may possibly mediate suppression of food intake in response to fasted HIE (**Figure 7**). Notably, the current study only examined neuronal changes 1-hour post-exercise, the time point immediately before changes in food intake were first observed, and changes in neuronal activity after exercise are rapid and transient (5–7). For example, it is unclear what is driving compensatory increases in food intake 6-12-hours after fasted exercise. To better understand the driving factors in post-exercise feeding, future studies into the time course of neuronal activity changes would be valuable.

Overall, our results identify a novel role for pre-exercise energy status to differentially modify post-exercise feeding behavior and hypothalamic neuron activity, which may explain the significant variability in results from studies investigating exercise as a weight loss intervention. Many other factors are also likely involved in mediating differences in post-exercise feeding, such as sex, training status, exercise volume, time of day, and type of exercise. For example, while our study utilized only male mice, female mice exhibit different regulatory mechanisms governing energy balance, including higher POMC expression (62), lower NPY expression (63), and a critical role for estrogen receptors in CNS control of feeding (64). Furthermore, since we investigated acute aerobic exercise, it is unclear if repeated aerobic exercise training or resistance training also have differential effects on CNS control of food intake. Lastly, to investigate the effects of exercise intensities on food intake, the current study did not control for total exercise volume or energy expenditure. It is possible that the observed effects of fasted HIE to decrease food intake and increase POMC^ARC^ neuron activity are due to increased exercise volume rather than intensity.

The effect of exercise on hypothalamic neuron activity is a relatively new focus of research and CNS control of feeding behavior is dynamic and complex. While many factors are likely involved, the current study identifies pre-exercise energy status as a novel variable that drastically modifies post-exercise food intake and appetite-controlling neurons. These findings could have implications when tailoring exercise programs to individual goals; for example, in clinical populations, fasted HIE may be beneficial to decreasing food intake and maximizing caloric deficit.

## Guarantor Statement 

HH is the guarantor of this work and, as such, has full access to all the data in the study and takes responsibility for its integrity and the accuracy of the analysis.

## Data Availability Statement

The raw data supporting the conclusions of this article will be made available by the authors, without undue reservation.

## Ethics Statement

The animal study was reviewed and approved by Institutional Animal Care and Use Committee of East Carolina University.

## Author Contributions 

TL conceptualization, methodology, validation, formal analysis, investigation, writing – original draft, writing – review and editing, and visualization. DS investigation, writing – review and editing. AC conceptualization, writing – review and editing. KF investigation. TN investigation. HH writing – review and editing, supervision, project administration, funding acquisition. All authors contributed to the article and approved the submitted version.

## Funding

The funding for this project was provided by East Carolina University start up, the National Institute of Diabetes and Digestive and Kidney Disease (DK121215) to HH.

## Conflict of Interest

The authors declare that the research was conducted in the absence of any commercial or financial relationships that could be construed as a potential conflict of interest.

## Supplementary Material

Supplementary Figure 1 Acute moderate intensity treadmill exercise suppresses food intake in fasted DIO male mice. **(A)** Timeline of cumulative food intake, **(B)** 48 hour cumulative food intake, and **(C)** food intake by time intervals in fasted male mice in response to different acute treadmill exercise intensities (n=11). Data represented as mean ± SEM. $ indicates p<0.05 moderate intensity vs. sedentary.

Supplementary Figure 2 ARC pSTAT3^tyr705^ and pERK^thr202/tyr204^ are unchanged 1 hour after high intensity treadmill exercise in fasted male mice. **(A)** Representative inverted DAB images of pSTAT3^tyr705^ (white) colocalized with NPY/AgRP^ARC^ (green) and POMC^ARC^ neurons (red) in fasted male mice 1 hour after sedentary trials or high intensity treadmill exercise. **(B)** Colocalization with NPY/AgRP^ARC^ neurons and **(C)** Colocalization with POMC neurons. **(D)** Representative DAB images of pERK^thr202/tyr204^ (black) in the ARC. **(E)** Total ARC pERK^thr202/tyr204^ (n=4-5/group). 3V = Third ventricle; Scale bar = 50um. Data represented as mean ± SEM.

Supplementary Figure 3 Fed mice exhibit elevated POMC^ARC^ neuron activity and reduced food intake. **(A)** Comparison of cFOS colocalized with POMC^ARC^ neurons in sedentary fasted mice vs. sedentary fed mice (original data presented in **Figure 6**). **(B)** Comparison of cFOS colocalized with NPY/AgRP^ARC^ neurons in sedentary fasted mice vs. sedentary fed mice (original data presented in **Figures 3** and **6**). **(C)** Comparison of cumulative food intake in sedentary fasted mice vs. sedentary fed mice (original data presented in **Figures 1** and **4**).

Supplementary Figure 4 High intensity treadmill exercise has no effects on TH^ARC^ neuron activity. **(A)** Representative images of cFOS (green) colocalized with TH^ARC^ neurons (red) in fed male mice 1 hour after sedentary trials or high intensity treadmill exercise. **(B)** Total and cFOS-expressing TH^ARC^ neurons and **(C)** Percent active TH^ARC^ neurons (n=5-6/group).
