# Supplementary figures and images for: Energy Status Differentially Modifies Feeding Behavior and POMCARC Neuron Activity After Acute Treadmill Exercise in Untrained Mice
Source: Front Endocrinol (Lausanne). 2021 Jun 18;12:705267. doi: 10.3389/fendo.2021.705267 (PMC8253227; doi:10.3389/fendo.2021.705267)

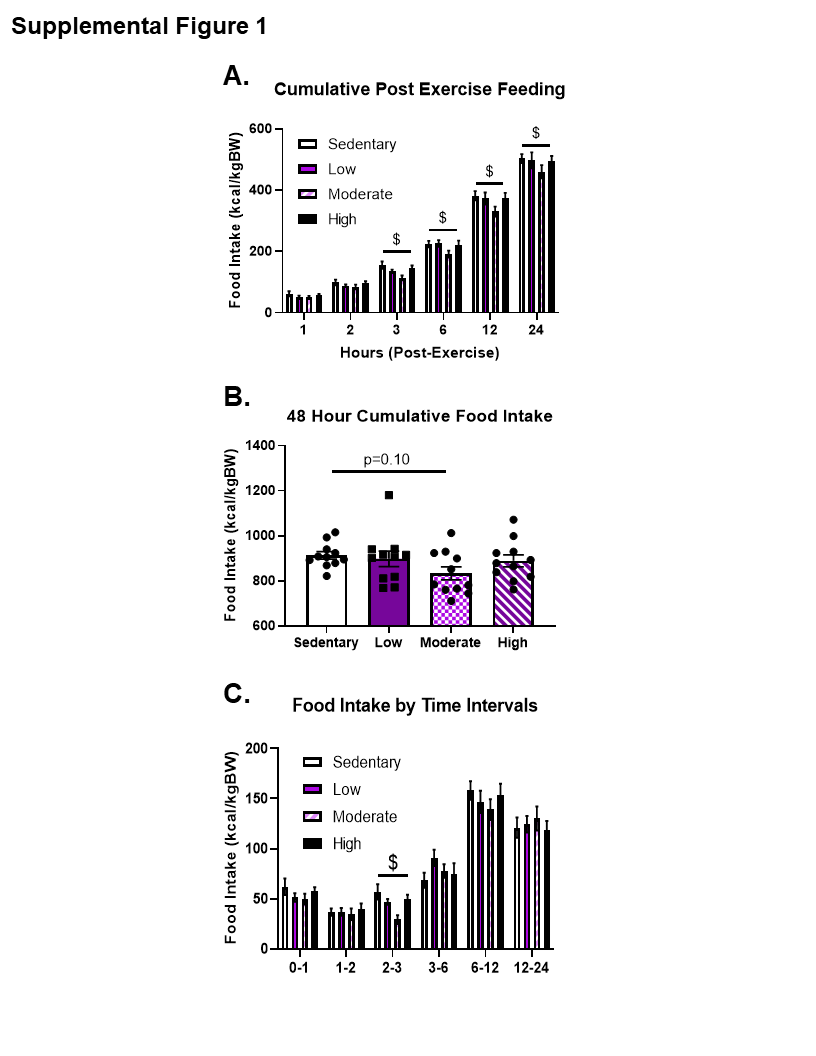

Supplement: Supplementary Figure 1 — Acute moderate intensity treadmill exercise suppresses food intake in fasted DIO male mice. (A) Timeline of cumulative food intake, (B) 48 hour cumulative food intake, and (C) food intake by time intervals in fasted male mice in response to different acute treadmill exercise intensities (n=11). Data represented as mean ± SEM. $ indicates p<0.05 moderate intensity vs. sedentary. [file Image_1.tif]

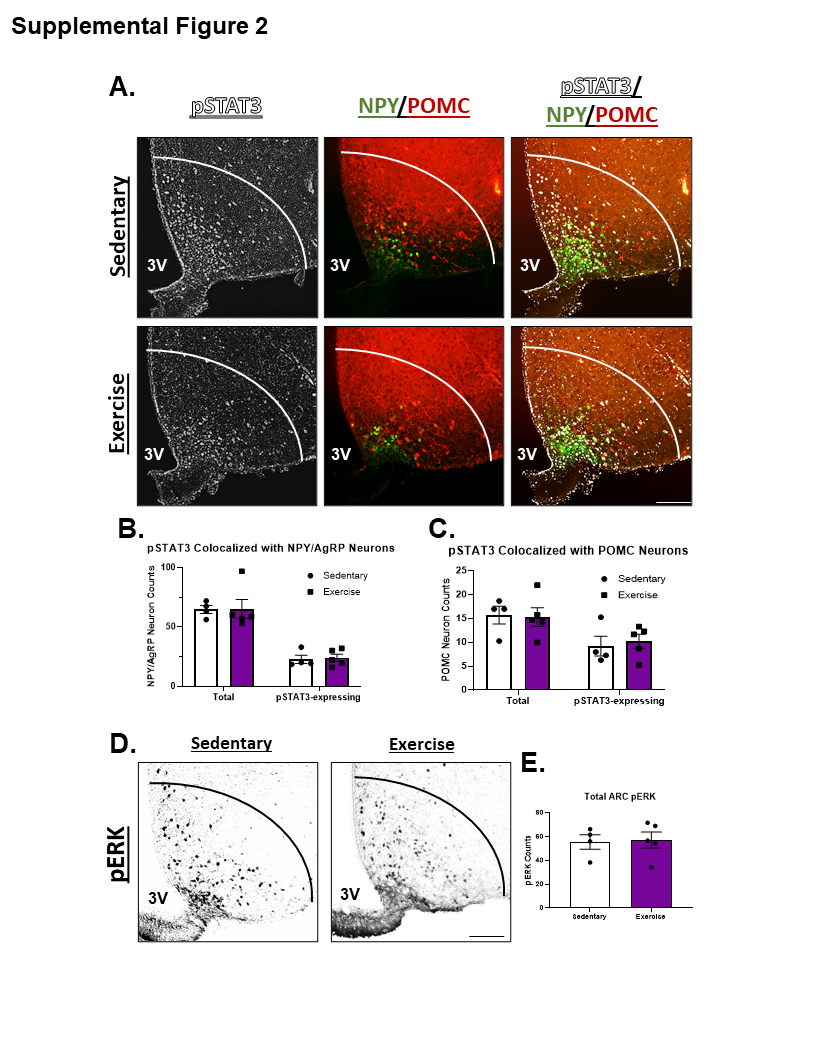

Supplement: Supplementary Figure 2 — ARC pSTAT3tyr705 and pERKthr202/tyr204 are unchanged 1 hour after high intensity treadmill exercise in fasted male mice. (A) Representative inverted DAB images of pSTAT3tyr705 (white) colocalized with NPY/AgRPARC (green) and POMCARC neurons (red) in fasted male mice 1 hour after sedentary trials or high intensity treadmill exercise. (B) Colocalization with NPY/AgRPARC neurons and (C) Colocalization with POMC neurons. (D) Representative DAB images of pERKthr202/tyr204 (black) in the ARC. (E) Total ARC pERKthr202/tyr204 (n=4-5/group). 3V = Third ventricle; Scale bar = 50um. Data represented as mean ± SEM. [file Image_2.tif]

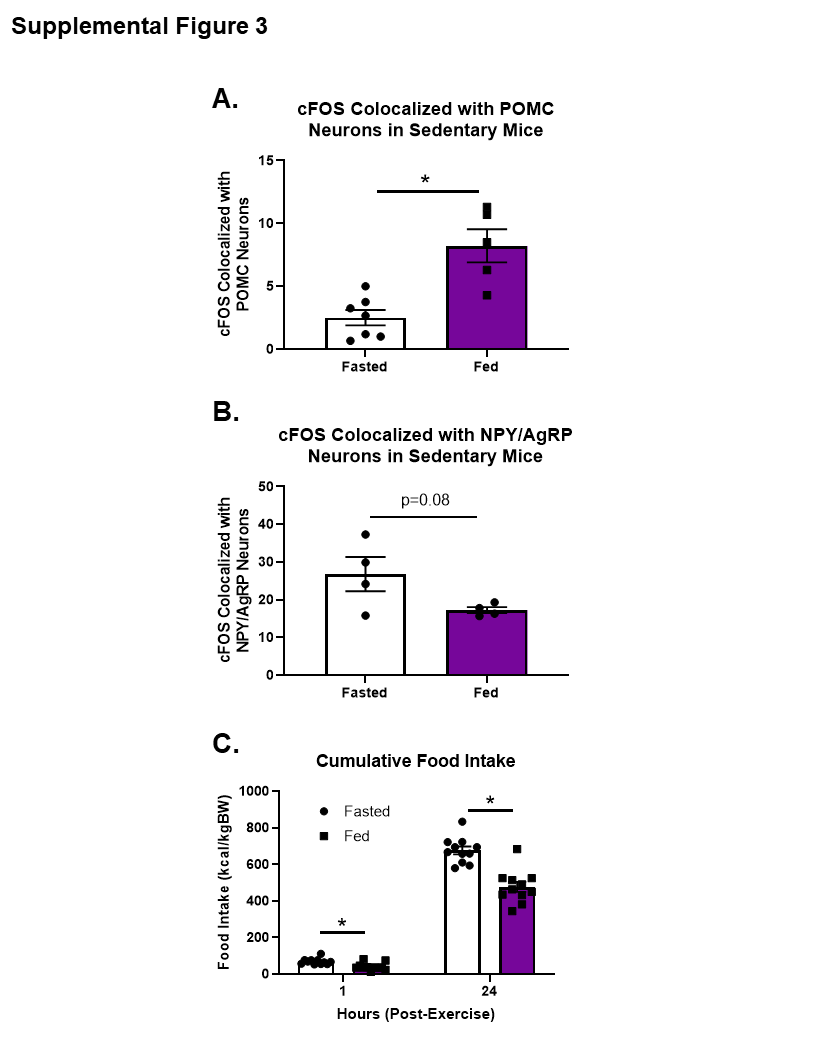

Supplement: Supplementary Figure 3 — Fed mice exhibit elevated POMCARC neuron activity and reduced food intake. (A) Comparison of cFOS colocalized with POMCARC neurons in sedentary fasted mice vs. sedentary fed mice (original data presented in Figure 6 ). (B) Comparison of cFOS colocalized with NPY/AgRPARC neurons in sedentary fasted mice vs. sedentary fed mice (original data presented in Figures 3 and 6 ). (C) Comparison of cumulative food intake in sedentary fasted mice vs. sedentary fed mice (original data presented in Figures 1 and 4 ). [file Image_3.tif]

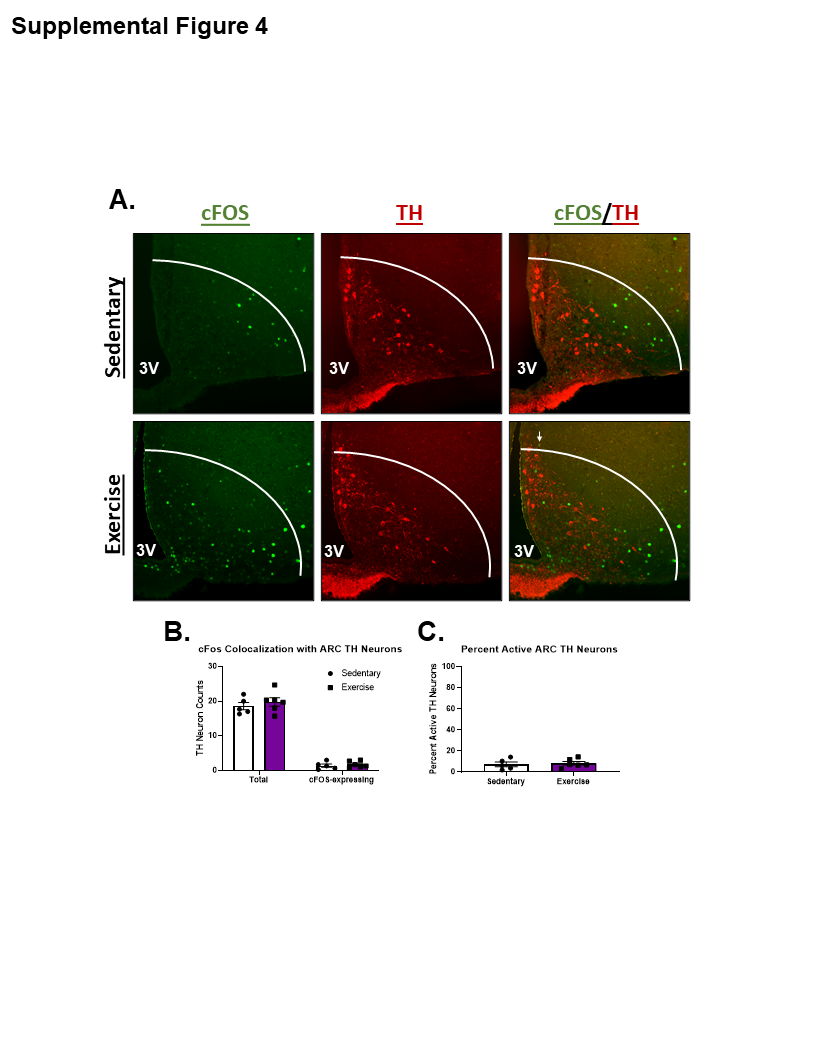

Supplement: Supplementary Figure 4 — High intensity treadmill exercise has no effects on THARC neuron activity. (A) Representative images of cFOS (green) colocalized with THARC neurons (red) in fed male mice 1 hour after sedentary trials or high intensity treadmill exercise. (B) Total and cFOS-expressing THARC neurons and (C) Percent active THARC neurons (n=5-6/group). [file Image_4.tif]
